# The Association of Diabetic Retinopathy and Cardiovascular Disease: A 13-Year Nationwide Population-Based Cohort Study

**DOI:** 10.3390/ijerph18158106

**Published:** 2021-07-30

**Authors:** Chin-Yuan Hsu, Chee-Ming Lee, Kuan-Yu Chou, Chia-Yi Lee, Hung-Chi Chen, Jeng-Yuan Chiou, Min-Yen Hsu

**Affiliations:** 1 Department of Health Policy and Management, Chung Shan Medical University, Taichung 40201, Taiwan; allen224c@yahoo.com.tw (C.-Y.H.); drchiou@hotmail.com (J.-Y.C.); 2 Department of Ophthalmology, Chung Shan Medical University Hospital, Taichung 40201, Taiwan; jimboy85@gmail.com (C.-M.L.); j830616g@gmail.com (K.-Y.C.); 3 School of Medicine, Chung Shan Medical University, Taichung 40201, Taiwan; 4 Department of Ophthalmology, Show Chwan Memorial Hospital, Changhua 50093, Taiwan; ao6u.3msn@hotmail.com; 5 Department of Ophthalmology, Chang Gung Memorial Hospital, Linkou 33305, Taiwan; mr3756@cgmh.org.tw; 6 Department of Medicine, College of Medicine, Chang Gung University, Taoyuan 33302, Taiwan; 7 Center for Tissue Engineering, Chang Gung Memorial Hospital, Linkou 33305, Taiwan; 8 Biotechnology Center, National Chung Hsing University, Taichung 40227, Taiwan

**Keywords:** diabetic retinopathy, cardiovascular disease, nationwide population-based epidemiological study

## Abstract

Objectives: Previous studies have demonstrated that patients with diabetic retinopathy (DR) have a higher prevalence of risk factors known to be associated with cardiovascular disease (CVD)**.** We hypothesized that patients with more severe DR could have a higher relative risk of CVD. Methods: To test this hypothesis, we used the National Health Insurance Research Database (NHIRD) to evaluate whether associations exist between DR and CVD. The data for this nationwide population-based retrospective cohort study were obtained from the NHIRD in Taiwan from 2001 to 2013. The assessed study outcome used was the incidence and other statistical analyses of CVD in patients with DR during a 13-year follow-up period. Results: Our findings obtained from 2001 to 2013 suggest that the incidence rates of CVD are 2.026 times that of diabetes mellitus (DM) without DR (95% C.I. = 1.876–2.187) and 2.75 times that of DM with DR (95% C.I. = 2.487–3.04) compared with the Non-DM group. Conclusion: The relative risk of CVD in DR was greater than that in the Non-DM group for both men and women. Targeted monitoring of DM, especially the co-existence of diabetic retinopathy, is of utmost importance in the clinical care of the DM population.

## 1. Introduction

Diabetic retinopathy (DR) is a chronic eye disease in diabetic patients that impairs the patient’s vision in an imperceptible way and it is one of the major causes of vision loss. The pathogenetic mechanism of DR remains obscure. Hyperglycemia is a major factor that causes damage to the microvascular system and results in retinal tissue destruction.

In the U.S., DR is the leading cause of vision loss in adults aged 20 to 64 years. A study that encompassed 35 worldwide studies conducted in the time span from 1980 to 2008 suggests that the global prevalence rate of any DR and proliferative diabetic retinopathy (PDR) in diabetic patients are 35.4% and 7.5%, respectively [1]. The prevalence rate of DR in the U.S. is high with 4.1 million Americans—that is, 1 in every 29—aged 40 years and above suffering from DR. The prevalence rate of DR is also expected to grow substantially in 2020 in consideration of population aging in the U.S. [2]. According to a survey conducted by the Diabetes Health Promotion Organization in Taiwan in 2011, the prevalence rate of DR is 26.5% among diabetes mellitus (DM) patients. There are two types of DR: non-proliferative diabetic retinopathy (NPDR) and proliferative diabetic retinopathy (PDR). The ocular signs of NPDR include microaneurysms, retinal hemorrhages, hard exudates, cotton wool spots, venous beading and intraretinal microvascular abnormalities. In PDR, the neovascularization of the retina, optic disc or iris can be noted due to advanced stages of retinal ischemia. According to the 1999 National Health Interview Survey in the U.S., the prevalence rates of NPDR and PDR in people over 40 years old are 40.3% and 8.2%, respectively [2].

It has been observed that one-third of the diabetic population is correlated with the increased risk of life-threatening systemic vascular complications (including strokes, coronary heart disease and heart failure) [3]. The Atherosclerosis Risk in Communities (ARIC) study conducted between 1993 and 1996 suggests that the 3-year incidence of any type of DR is 3.8% [4]. Our study is a nationwide population-based epidemiological study that aims to investigate the risk of cardiovascular disease (CVD) among DM patients using a national healthcare database.

## 2. Materials and Methods

### 2.1. Sources of Data

The study is a retrospective cohort study based in part on data from the National Health Insurance Research Database (NHIRD). Since the implementation of the National Health Insurance in 1995, over 99% of the 23 million population in Taiwan were covered by health insurance in 2018, making the NHIRD a representative source of empirical data for medical research. Established by the National Health Research Institutes (NHRI) in Taiwan, the NHIRD encompasses data on the diagnosis of both outpatients’ and hospitalized patients’ disease as well as medication, the disposal of patients and expense. In recent years, the Health and Welfare Data Science Center of the Ministry of Health and Welfare was established to centralize and manage research data on health and welfare, which includes the Longitudinal Health Insurance Database 2005 (LHID 2005). The LHID 2005 randomly selects two million representative samples from the NHIRD and analyzes the samples using data on diabetes diagnoses. The individuals’ health insurance declaration during the time span from 1 January 2001 to 31 December 2013 was used as our data source.

**Figure 1:**
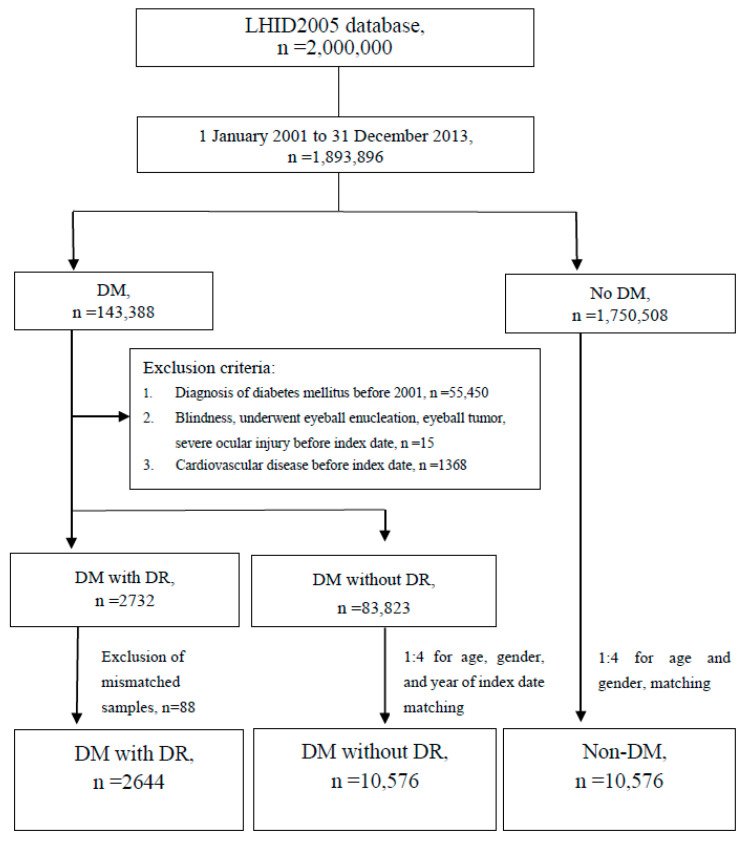
Study flowchart.

From 1 January 2001 to 31 December 2013, there were 143,388 people with a diagnosis of DM and 1,750,508 people without DM. Among the patients with DM, those who were diagnosed with DM before 2001, blind, underwent an eyeball enucleation, had an eyeball tumor and had a severe ocular injury before being diagnosed with DM were excluded. This resulted in a total of 86,555 people with DM to be analyzed. Among these patients, 2732 were diagnosed with DR and 83,823 patients without DR. Among the patients with DR, 1326 were diagnosed with NPDR and 1318 were diagnosed with PDR. The study population was classified into three groups for comparison: DM with DR, DM without DR and Non-DM. After the exclusion of mismatched samples, there were 2644 patients in the group of DM with DR. Through pair matching on age, gender and year of index date at a ratio of 1:4, there were 10,576 people in the group of DM without DR. Through pair matching on age and gender at a ratio of 1:4, there were 10,576 people in the group of Non-DM. The three groups were investigated for the diagnosis of newly occurred cardiovascular events during the study period. The study protocol is shown in Figure 1.

### 2.2. Definitions of Primary Events

Our definitions of primary events encompassed various classifications of ischemic heart diseases, coronary artery diseases, congestive heart failures, cardiomyopathies, basilar artery occlusions/stenosis and cerebral ischemia/infarctions. To eliminate the possibility of underestimating the incidence of primary events, we did not exclude the diagnostic coding associated with systemic diseases such as rheumatic diseases, hypertension or renal diseases. To eliminate the confounding effect of these systemic diseases on primary events, our statistical results were adjusted by comorbidities such as rheumatic diseases, renal diseases and chronic obstructive pulmonary disease (COPD). The definitions of the cardiovascular diseases (CVDs) selected by the study according to the International Classification of Diseases, Ninth Revision, Clinical Modification (ICD-9-CM) are as follows: ischemic heart diseases (ICD-9 codes: 410.x, 412.x, 414.0, 414.0x, 414.2, 414.3, 414.4, 414.8, 414.9), congestive heart failure (ICD-9 codes: 398.91, 402.01, 402.11, 402.91, 404.01, 404.03, 404.11, 404.13, 404.91, 404.93, 425.4–425.9, 428.x) and ischemic strokes (ICD-9 codes: 433–435). The date of the first diagnosis of the CVD was set as the date of the occurrence of the event (Table 1).

### 2.3. Statistical Analysis

Our study examined the basic characteristics in our study groups including age, gender, comorbidities, the occurrence of eye diseases, concomitant medications, the duration of diabetes and the type of retinopathy. The correlation of DR and the type of retinopathy with the risk of the occurrence of CVD were investigated. The descriptive analysis of the number of counts and proportion was conducted. The chi-squared test was then used to examine the differences of categorical variables between groups. The incidence density rate and its 95% confidence interval were calculated following the Poisson assumption. A multiple Cox proportional hazard regression for the estimation of adjusted hazard ratios on any CVD and population analysis were conducted to examine the correlation between DR and the occurrence of CVD. The Bonferroni correction was used to conduct a multiple comparison. All statistical analyses were performed by using SAS^®^ software version 9.4 (SAS Institute Inc., Cary, NC, USA). A significance level of 0.05 with the two tailed test was used in this study.

## 3. Results

### 3.1. Demographics

Table 2 shows the basic characteristics in our study groups. In the DR group, the age group of 40–59 years occupied the highest proportion of people with a total of 1519 (57.45%) and the age group of 60–79 years had the second highest proportion of people with a total of 917 (34.68%). The number of females was 1406 (53.18%) and the number of males was 1238 (46.82%). Given the age and sex matching design, the distributions of age and sex in the Non-DM group, the DM without DR group and the DM with DR group were identical.

The difference in comorbidities and the concomitant drug between the Non-DM group and the DM with DR is represented by *p*-value 1 (p1) and the difference in comorbidity between the DM with DR and DM without DR group is represented by *p*-value 2 (p2). A statistically significant difference was observed between the Non-DM group and the DM with DR group in the prevalence rate of hypertension, hyperlipidemia, chronic pulmonary disease, renal disease, hepatitis and liver cirrhosis, alcoholic liver disease, gout, allergic rhinitis, keratopathy, dry eye syndrome, uveitis, glaucoma, age-related macular degeneration and cataracts. There was also a statistically significant difference observed between the DM with DR and DM without DR groups in the prevalence rate of hypertension, dementia, chronic pulmonary disease, rheumatic disease, renal disease, hepatitis and liver cirrhosis, alcoholic liver disease, gout, atopic dermatitis, allergic rhinitis, keratopathy, dry eye syndrome, uveitis, glaucoma, age-related macular degeneration and cataracts.

As for the concomitant drug, a significant difference was observed in the usage proportion of systemic steroids, topical steroids, antipsychotics, hypolipidemics and hypoglycemics observed between the Non-DM group and the DM with DR group. A significant difference was also observed in the usage of systemic steroids, topical steroids, antipsychotics, hypolipidemics and hypoglycemics between the DM with DR and DM without DR groups.

### 3.2. Analysis of the Incidence of CVD

Table 3 shows the analysis results of the incidence of any CVD. The incidence of CVD of the Non-DM group was 16.07 (95% C.I. = 15.11–17.1) per 10,000 person-months. The incidence of CVD of the DM without DR group was 32.78 (95% C.I. = 31.33–34.3) per 10,000 person-months. The incidence of CVD of the DM with DR group was 44.64 (95% C.I. = 41.24–48.32) per 10,000 person-months. In terms of the risk ratio of any CVD, the Bonferroni adjusted risk ratios of DM with DR and DM without DR compared with the Non-DM groups were 2.75 (95% C.I. = 2.433–3.109) and 2.026 (95% C.I. = 1.845–2.225), respectively, both of which were statistically significant.

### 3.3. Adjusted Hazard Ratios of CVD among All Study Groups

Table 4 shows the adjusted hazard ratios of CVD compared among the groups. The hazard ratios of the Non-DM group compared with the DM group and DM with DR compared with the DM without DR groups were 0.573 (95% C.I. = 0.524–0.627) and 1.215 (95% C.I. = 1.093–1.350), respectively, both of which were statistically significant.

In terms of age, in comparison with the age group of 40–59 years, the people in the age group of 80 years and above had the highest hazard ratio, which was 2.220 (95% C.I. = 1.745–2.823) and was statistically significant. The age group of 60–79 years had the second highest hazard ratio, which was 1.616 (95% C.I. = 1.499–1.743) and was statistically significant. The hazard ratio of the age group below 40 years was 0.569 (95% C.I. = 0.463–0.700) and was statistically significant.

In terms of sex, in comparison with the female group, the male group had a hazard ratio of 1.279 (95% C.I. = 1.194–1.372) that was statistically significant. In terms of comorbidity, the hazard ratio of hypertension was 1.761 (95% C.I. = 1.633–1.900) and was statistically significant. The hazard ratio of dementia was 1.547 (95% C.I. = 1.182–2.204) and was statistically significant. The hazard ratio of chronic pulmonary disease was 1.177 (95% C.I. = 1.089–1.273) and was statistically significant. The hazard ratio of renal disease was 1.251 (95% C.I. = 1.118–1.401) and was statistically significant. The hazard ratio of hepatitis and liver cirrhosis was 0.880 (95% C.I. = 0.815–0.950) and was statistically significant. The hazard ratio of cataracts was 1.154 (95% C.I. = 1.053–1.265) and was statistically significant.

In terms of concomitant medication, the hazard ratio of systemic steroids was 1.204 (95% C.I. = 1.109–1.307) and was statistically significant. The hazard ratio of topical steroids was 1.115 (95% C.I. = 1.022–1.216) and was statistically significant. The hazard ratio of antipsychotics was 1.316 (95% C.I. = 1.138–1.521) and was statistically significant. The hazard ratio of antiarrhythmics was 2.491 (95% C.I. = 1.633–3.801) and was statistically significant. The hazard ratio of hypoglycemics was 0.878 (95% C.I. = 0.809–0.952) and was statistically significant.

### 3.4. Adjusted Hazard Ratios of CVD among the Diabetic Population

Table 5 shows the analysis results of a more in-depth exploration of the potential risk factors in the occurrence of CVD in the DM population. In the analysis of the history of DM, with the patients with a history of DM of less than 2 years categorized as the reference group, the hazard ratio of a history of diabetes of 2–5 years was 0.788 (95% C.I. = 0.717–0.866) and the hazard ratio of a history of DM of 5 years and above was 0.779 (95% C.I. = 0.690–0.879), both of which were statistically significant.

In order to compare the influence on the risk of CVD in different types of retinopathy, the DM without DR group was used as the reference. The hazard ratio of PDR was 1.612 (95% C.I. = 1.403–1.851) and was statistically significant. However, the hazard ratio of NPDR was 0.940 (95% C.I. = 0.816–1.084) and was not statistically significant.

In terms of age, with the age group of 40–59 years with the risk of CVD as the control group, the hazard ratio of those under 40 years of age was 0.605 (95% C.I. = 0.482–0.759) and was statistically significant. The hazard ratio of those between 60 and 79 years of age was 1.549 (95% C.I. = 1.416–1.694) and was statistically significant. The hazard ratio of those of 80 years and above was 2.056 (95% C.I. = 1.535–2.753) and was statistically significant.

In terms of sex, with the risk of CVD in females as the standard, the hazard ratio of CVD in males was 1.311 (95% C.I. = 1.207–1.424) and was statistically significant. In terms of comorbidity, the hazard ratio of hypertension was 1.607 (95% C.I. = 1.474–1.755) and was statistically significant. The hazard ratio of chronic pulmonary disease was 1.142 (95% C.I. = 1.04–1.253) and was statistically significant. The hazard ratio of renal disease was 1.283 (95% C.I. = 1.133–1.454) and was statistically significant. The hazard ratio of hepatitis and liver cirrhosis was 0.846 (95% C.I. = 0.774–0.924) and was statistically significant. The hazard ratio of cataracts was 1.181 (95% C.I. = 1.064–1.312) and was statistically significant.

In terms of concomitant medication, the hazard ratio of systemic steroids was 1.194 (95% C.I. = 1.085–1.314) and was statistically significant. The hazard ratio of antipsychotics was 1.298 (95% C.I. = 1.104–1.527) and was statistically significant. The hazard ratio of antiarrhythmics was 2.072 (95% C.I. = 1.1239–3.465) and was statistically significant. The hazard ratio of hypoglycemics was 0.882 (95% C.I. = 0.812–0.959) and was statistically significant.

### 3.5. Stratified Analysis of CVD among All Study Groups

Table 6 shows the hazard ratios of CVD in the DM with DR and Non-DM groups with the DM without DR group as the reference. The hazard ratio of CVD of the DM with DR group was 1.215 (95% C.I. = 1.068–1.382) and was statistically significant. The hazard ratio of CVD of the Non-DM group was 0.537 (95% C.I. = 0.514–0.639) and was statistically significant.

The comparison of the risks of ischemic heart disease showed that the hazard ratios of ischemic heart disease of the DM with DR group and Non-DM group with respect to the DM without DR group were 1.194 (95% C.I. = 1.004–1.421) and 0.614 (95% C.I. = 0.531–0.710), respectively, both of which were statistically significant.

The comparison of the risks of congestive heart failure showed that the hazard ratios of congestive heart failure of the DM with DR group and Non-DM group with respect to the DM without DR group were 1.915 (95% C.I. = 1.376–2.665) and 0.642 (95% C.I. = 0.464–0.889), respectively, both of which were statistically significant.

The comparison of the risks of an ischemic stroke showed that the hazard ratios of an ischemic stroke of the DM with DR group and Non-DM group with respect to the DM without DR group were 2.003 (95% C.I. = 1.331–3.015) and 0.675 (95% C.I. = 0.462–0.986), respectively, both of which were statistically significant.

### 3.6. Stratified Analysis of CVD among the Diabetic Population

Table 7 compares the hazard ratios of CVD of the NPDR group and those of the PDR group with the DM without DR group as the reference. In the PDR group, the hazard ratios of CVD compared with the DM without DR group were as follows: any CVD (1.612 (95% C.I. = 1.361–1.909)), ischemic heart disease (1.545 (95% C.I. = 1.231–1.939)), congestive heart failure (3.482 (95% C.I. = 2.334–5.195)) and an ischemic stroke (3.802 (95% C.I. = 2.261–6.394)), all of which were statistically significant. However, in the NPDR group, none of the risks of any CVD was significantly higher than any of those of the DM without DR group.

## 4. Discussion

The study was a nationwide population-based retrospective cohort study that examined the correlation between DR and the occurrence of CVD by using the NHIRD, which has a coverage rate of over 99%. The findings obtained from the inspection period from 2001 to 2013 suggest that the incidence rates of CVD were 2.026 times that of DM without DR (95% C.I. = 1.845–2.225) and 2.750 times that of DM with DR (95% C.I. = 2.433–3.109) compared with the Non-DM group, as shown in Table 3. The findings correspond with the findings of Xie [5] that the overall risk of death and/or CVD in patients with any type of DR is twice that of those without DR.

Given the relatively large number of risk factors for CVD in DR in the study, after the adjustment in variables such as comorbidity, the Non-DM group showed a lower risk of CVD with the adjusted hazard ratios being 0.573 (95% C.I. = 0.524–0.627). The DM with DR group showed a higher risk of CVD with the adjusted hazard ratios being 1.215 (95% C.I. = 1.093–1.350) (Table 4). Such findings also correspond with the hypothesis in our study that patients with diabetic retinopathy have a higher risk of CVD.

Furthermore, in Table 2, hypertension (61.54%), hyperlipidemia (59.57%) and cataracts (53.59%) all reached a significant level. One of the reasons for such an increase is the frequency of the risk factors for CVD such as hypertension, dyslipidemia and coagulopathy. There is a strong epidemiological correlation between hypertension caused by diabetes and the adverse consequences of diabetes. The epidemiological study from the UK Prospective Diabetes Study (UKPDS) suggests that with a decrease in the mean systolic pressure of 10 mmHg, the risk of any diabetes-related complication decreases by 12%, the risk of death from diabetes decreases by 15%, the risk of myocardial infarction decreases by 11% and the risk of a microvascular complication decreases by 13%. Do et al.’s [6] study also finds that lowering blood pressure is effective in preventing DR for 4–5 years. Tada et al.’s [7] most recent findings also explain the correlation between triglycerides in serum and the first occurrence of CVD in diabetic patients with a high risk of hypercholesterolemia and retinopathy.

In Cheung et al.’s [8] study on DR and coronary artery disease, 214 (14.7%) of the participants were diagnosed with DR. A follow-up examination of an average of 7–8 years showed that 209 (13.7%) of the patients were diagnosed with coronary artery disease. The patients suffering from a low-to-medium level of NPDR had a higher risk of coronary artery disease (HR, 1.69; 95% C.I. 1.17–2.97) and strokes (HR, 2.69; 95% C.I. 1.03–4.86). The presence of a retinal hemorrhage or microaneurysm was correlated with the risk of coronary artery disease (HR, 1.63; 95% C.I. 1.04–2.56), which corresponded with the number in our finding (that is, 1.194 times (1.036–1.377)) and verifies the fact that the presence of DR implies a higher risk of coronary heart disease. Moreover, Cheung et al.’s [9] findings also showed that patients with retinopathy are 2.5 times more likely to be diagnosed with HF than patients without retinopathy (HR, 2.71; 95% C.I. 1.46–5.05). Zhang et al.’s [10] study showed a higher risk of heart failure (RR 2.68, 95% C.I. 1.34–5.36 in T1D; RR 2.25, 95% C.I. 1.91–2.65 in T2D). The findings corresponded with the DR in the DM group in our study; that is, 1.915 (95% C.I. 1.461–2.51).

Zhu et al.’s [10] study compared patients with DR with those without DR and found that DR was correlated with a significant increase in the risk of strokes (RR = 1.74, 95% C.I. 1.35–2.24). The finding corresponded with the number in our finding; that is, 2.003 (95% C.I. 1.433–2.799).

According to the recent systematic review and meta-analysis on diabetic macular edema (DME), PDR and CVD, in comparison with patients without DME or PDR, patients with DME or PDR have a high risk of CVD and fatal CVD, which corresponded with our findings [5]. In other words, the most significant time correlation can be observed soon after the occurrence of hypoglycemia. The risks of MACE, death from CVD, death from non-CVD factors or all-cause mortality may increase during the shorter follow-up investigation period after severe hypoglycemia. Given that patients with T2DM, DME or PDR are more likely to suffer CVD, these patients should undergo more intensive follow-ups in order to prevent CVD. Apart from the post-hoc analysis of the randomized controlled trials, some epidemiological evidence and studies have also demonstrated the time correlation between the occurrence of PDR and that of CVD. In van Hecke et al.’s [11] study, the researchers investigated the follow-up of 2237 patients with type 1 diabetes from 31 regions of 16 European countries after 6–8 years of their diagnosis and examined the death rate and incidence of CVD. After a follow-up of 7.9 years, 64 of the patients were deceased and 128 of the patients were diagnosed with CVD. The study showed that the all-cause death rate of patients with type 1 diabetes and NPDR or PDR was correlated with a higher risk of CVD. Such a correlation could be substantially clarified by the presence of the risk factors for CVD. It was implied that, apart from the correlation with PDR, there are other mutual mechanisms involved [11]. Such findings also correspond with our analysis results. A higher level of severity of retinopathy over time indicates a higher risk of CVD. The level of severity of retinopathy and the development of the disease are determinant factors for CVD. The retina can serve as a factor in metabolism and hemodynamics as well as an anatomic indicator of the effects of future CVD [12]. Our findings suggested that CVD was correlated with the duration of DM. A longer duration of DM would result in a higher risk of CVD. Furthermore, our findings also suggested that DM patients diagnosed with DR should prevent the risk of CVD through more intensive and frequent care and health management.

A study published by the Singapore Malay Eye Study suggests that age, blood pressure, ametropia and lens opacity have a significant impact on the fractal measurement of retinal blood vessels. A new assessment of retinal vascular optimality that combines fractals and caliber suggests a strong correlation with blood pressure. As a result, the quantitative analysis of retinal vasculature may provide an additional insight into microvascular structure and optimality [13]. Several major epidemiological studies have agreed on a correlation between the factors in systemic disease and the caliber of the retinal blood vessels. It has been particularly confirmed that an increase in systemic blood pressure is reflected in systemic arterial vasoconstriction of the retinal blood vessels [14]. The primary systemic determinant of a narrower caliber of retinal arteriole is higher blood pressure whereas those of a broader caliber of retinal venule include smoking, hypertension, systemic inflammation and obesity [15]. In other words, there is increasing evidence to suggest that the diameter of a retinal blood vessel carries information on not only retinal circulation but also systemic pathological changes. The changes in the diameter of a retinal blood vessel in particular have been proven to be useful in predicting coronary heart disease, strokes and the risk of death from strokes [16,17]. The summarized data from the Beaver Dam Eye Study and Blue Mountains Eye Study also suggest that a shorter diameter of an artery and a longer diameter of a retinal vein are correlated with an increase in the risk of death from strokes and that the diameter of a retinal blood vessel can be utilized to predict the risk of death from cardiovascular disease or strokes in the middle-aged population [18]. It is clear from the aforementioned that many have suggested that the diameter of the retinal blood vessel can serve as a predictor of conditions that involve other vascular beds (such as the heart or the cerebrum). The changes in the retinal and choroidal capillaries are closely linked to stenosis of the coronary arteries and their branches [19].

Although the declared data have been meticulously reviewed by health professionals, declaration procedures of the medical institutions and the National Health Insurance Administration, classification errors caused by coding errors, overdiagnosis or underdiagnosis may have occurred leading to an underestimation of the correlation between DR and CVD. The correlation between DR and the increase in the risk of CVD in Asians is also associated with a more extensive range of potential confounding factors. Further ascertainment of the population is required in a greater longitudinal study and cannot be verified via other effective methods. Although the data can provide detailed diagnostic data, they do not offer certain information about the patient such as family history, educational level, socioeconomic background, BMI, smoking behavior or the severity of the comorbidity. The NHIRD does not provide data on the inspection items in biochemical examinations such as platelets, INR, PT, aPTT, LDL, HDL, cholesterol, triglycerides, D-dimer and fibrinogen, all of which are significant potential confounding factors for CVD that could not be adjusted in our study. In addition, as the samples were collected from the Taiwanese population, in consideration of the regional differences in DR and epidemiology, the findings of the study may not apply to other countries, regions or races.

## 5. Conclusions

The relative risk of CVD events in DR was greater than that in the control group for both men and women as well as for age. The targeted monitoring and management of DM, especially the detection of diabetic retinopathy, should be based on the patient’s cardiovascular disease risk factors for investigation and management, which may be important in the clinical care of the DM population.

## Figures and Tables

**Table 1:** ICD-9-CM codes and diagnosis.

ICD-9	Diagnosis	ICD-9	Diagnosis
410.00–410.92	Myocardial infarction	402.91	Unspecified hypertensive heart disease with congestive heart failure
412	Old myocardial infarction	404.01	Malignant hypertensive heart and renal disease with congestive heart failure
414.00–414.05	Coronary atherosclerosis	404.03	Malignant hypertensive heart and renal disease with congestive heart failure and renal failure
414.2	Chronic total occlusion of coronary artery	404.11	Benign hypertensive heart and renal disease with congestive heart failure
414.3	Coronary atherosclerosis due to lipid rich plaque	404.13	Benign hypertensive heart and renal disease with congestive heart failure and renal failure
414.4	Coronary atherosclerosis due to calcified coronary lesion	404.91	Unspecified hypertensive heart and renal disease with congestive heart failure
414.8	Other specified forms of chronic ischemic heart disease	404.93	Unspecified hypertensive heart and renal disease with congestive heart failure and renal failure
414.9	Chronic ischemic heart disease, unspecified	425.4–425.9	Cardiomyopathies
398.91	Rheumatic heart failure (congestive)	428.0–428.9	Congestive heart failure
402.01	Malignant hypertensive heart disease with congestive heart failure	433.00–433.91	Occlusion and stenosis of basilar artery
402.11	Benign hypertensive heart disease with congestive heart failure	434.00–434.91	Cerebral infarction, unspecified
		435.0~435.9	Transient cerebral ischemia

**Table 2:** Demographics.

	DM with DR *n* = 2644	DM without DR *n* = 10,576	Non-DM *n* = 10,576	*p*-Value 1	*p*-Value 2
Age				1.0000	1.0000
< 40	175 (6.6%)	700 (6.6%)	700 (6.6%)		
40–59	1519 (57.5%)	6076 (57.5%)	6076 (57.5%)		
60–79	917 (34.7%)	3668 (34.7%)	3668 (34.7%)		
≥ 80	33 (1.3%)	132 (1.3%)	132 (1.3%)		
Gender				1.0000	1.0000
Male	1238 (46.8%)	4952 (46.8%)	4952 (46.8%)		
Female	1406 (53.2%)	5624 (53.2%)	5624 (53.2%)		
Comorbidity					
Hypertension	1627 (61.5%)	5994 (56.7%)	2651 (25.1%)	<0.0001	<0.0001
Hyperlipidemia	1575 (59.6%)	6299 (59.6%)	1846 (17.5%)	<0.0001	1.0000
Dementia	15 (0.6%)	118 (1.1%)	74 (0.7%)	0.9131	0.0230
Chronic pulmonary disease	566 (21.4%)	2790 (26.4%)	2049 (19.4%)	0.0378	<0.0001
Rheumatic disease	64 (2.4%)	340 (3.2%)	267 (2.5%)	1.0000	0.0676
Renal disease	316 (12.0%)	880 (8.3%)	380 (3.6%)	<0.0001	<0.0001
Hepatitis and liver cirrhosis	766 (29.0%)	4372 (41.3%)	2069 (19.6%)	<0.0001	<0.0001
Alcoholic liver disease	58 (2.2%)	350 (3.3%)	96 (0.9%)	<0.0001	0.0060
Gout	379 (14.3%)	2317 (21.9%)	1072 (10.1%)	<0.0001	<0.0001
Atopic dermatitis	74 (2.8%)	396 (3.7%)	336 (3.2%)	0.6313	0.0377
ENT allergies	403 (15.2%)	2046 (19.4%)	1782 (16.9%)	0.0931	<0.0001
Eye disease					
Keratopathy	280 (10.6%)	714 (6.8%)	640 (6.1%)	<0.0001	<0.0001
Dry eye syndrome	310 (11.7%)	805 (7.6%)	629 (6.0%)	<0.0001	<0.0001
Uveitis	144 (5.5%)	101 (1.0%)	75 (0.7%)	<0.0001	<0.0001
Glaucoma	213 (8.1%)	306 (2.9%)	221 (2.1%)	<0.0001	<0.0001
Age-related macular degeneration	130 (4.9%)	106 (1.0%)	98 (0.9%)	<0.0001	<0.0001
Cataracts	1417 (53.6%)	1726 (16.3%)	1267 (12.0%)	<0.0001	<0.0001
Retinopathy after vitrectomy	130 (4.9%)	19 (0.2%)	46 (0.4%)	<0.0001	<0.0001
Concomitant drug					
Systemic steroids	587 (22.2%)	2038 (19.3%)	1483 (14.0%)	<0.0001	0.0015
Topical steroids	1526 (57.7%)	1655 (15.7%)	1367 (12.9%)	<0.0001	<0.0001
Antipsychotics	166 (6.3%)	515 (4.9%)	285 (2.7%)	<0.0001	0.0067
Antiarrhythmics	11 (0.4%)	37 (0.4%)	23 (0.2%)	0.1428	1.0000
Hypolipidemics	848 (32.1%)	2558 (24.2%)	390 (3.7%)	<0.0001	<0.0001
Hypoglycemics	1984 (75.0%)	5105 (48.3%)	185 (1.8%)	<0.0001	<0.0001
Duration of DM (year)				-	1.0000
0	0 (0.0%)	0 (0.0%)	10,576 (100.0%)		
<2	1311 (49.6%)	5244 (49.6%)	0 (0.0%)		
2–5	749 (28.3%)	2996 (28.3%)	0 (0.0%)		
≥5	584 (22.1%)	2336 (22.1%)	0 (0.0%)		
Type of retinopathy				-	-
Non-retinopathic	0 (0.0%)	10,576 (100.0%)	0 (0.0%)		
NPDR	1326 (50.2%)	0 (0.0%)	0 (0.0%)		
PDR	1318 (49.9%)	0 (0.0%)	0 (0.0%)		

p1: comparison between DM with DR with Non-DM; p2: comparison between DM with DR with DM without DR. p1 and p2 were all Bonferroni adjusted for multiple comparisons; DM: diabetes mellitus; DR: diabetic retinopathy; Non-DM: non-diabetic mellitus; NPDR: non-proliferative diabetic retinopathy; PDR: proliferative diabetic retinopathy.

**Table 3:** Incidence of cardiovascular disease in the examined groups.

	DM with DR *n* = 2644	DM without DR *n* = 10,576	Non-DM *n* = 10,576
Observed person-months	136,873	571,430	627,706
Newly diagnosed CVD	611	1873	1009
Incidence * (95% C.I.)	44.64 (41.24–48.32)	32.78 (31.33–34.30)	16.07 (15.11–17.10)
Crude relative risk (95% C.I.)	2.750 (2.487–3.040)	2.026 (1.876–2.187)	(Reference)
Bonferroni adjusted relative risk (95% C.I.)	2.750 (2.433–3.109)	2.026 (1.845–2.225)	(Reference)

* Incidence rate per 10,000 person-months; DM: diabetes mellitus; DR: diabetic retinopathy; Non-DM: non-diabetes mellitus; CVD: cardiovascular disease.

**Table 4:** Adjusted hazard ratios of CVD among all study groups.

Variable	aHR (95% C.I.)
Studied group	
Non-DM vs. DM	0.573 (0.524–0.627) *
DM with DR vs. DM without DR	1.215 (1.093–1.350) *
Age (reference: 40–59)	
<40	0.569 (0.463–0.700) *
60–79	1.616 (1.499–1.743) *
≥80	2.220 (1.745–2.823) *
Sex (reference: female)	
Male	1.279 (1.194–1.372) *
Comorbidity	
Hypertension	1.761 (1.633–1.900) *
Hyperlipidemia	0.983 (0.907–1.065)
Dementia	1.547 (1.182–2.024) *
Chronic pulmonary disease	1.177 (1.089–1.273) *
Rheumatic disease	1.184 (0.988–1.419)
Renal disease	1.251 (1.118–1.401) *
Hepatitis and liver cirrhosis	0.880 (0.815–0.950) *
Alcoholic liver disease	1.109 (0.870–1.413)
Gout	1.057 (0.968–1.154)
Atopic dermatitis	1.005 (0.834–1.212)
ENT allergies	0.939 (0.856–1.030)
Eye disease	
Keratopathy	0.935 (0.816–1.072)
Dry eye syndrome	0.989 (0.868–1.126)
Uveitis	1.063 (0.819–1.379)
Glaucoma	1.023 (0.864–1.211)
Age-related macular degeneration	0.925 (0.721–1.188)
Cataracts	1.154 (1.053–1.265) *
Retinopathy after vitrectomy	1.136 (0.838–1.540)
Concomitant medication	
Systemic steroids	1.204 (1.109–1.307) *
Topical steroids	1.115 (1.022–1.216) *
Antipsychotics	1.316 (1.138–1.521) *
Antiarrhythmics	2.491 (1.633–3.801) *
Hypolipidemics	1.069(0.974–1.174)
Hypoglycemics	0.878 (0.809–0.952) *

* *p*-value < 0.05; aHR: adjusted hazard ratios; Non-DM: non-diabetes mellitus; DM: diabetes mellitus; DR: diabetic retinopathy.

**Table 5:** Adjusted hazard ratios of CVD among the diabetic population.

Variable	aHR (95% C.I.)
Duration of diabetes (year) (reference: <2)	
2–5	0.788 (0.717–0.866) *
≥5	0.779 (0.690–0.879) *
Type of retinopathy	
NPDR vs. DM without DR	0.940 (0.816–1.084)
PDR vs. DM without DR	1.612 (1.403–1.851) *
Age (reference: 40–59)	
<40	0.605 (0.482–0.759) *
60–79	1.549 (1.416–1.694) *
≥80	2.056 (1.535–2.753) *
Sex (reference: female)	
Male	1.311 (1.207–1.424) *
Comorbidity	
Hypertension	1.607 (1.471–1.755) *
Hyperlipidemia	0.933 (0.853–1.021)
Dementia	1.371 (0.983–1.912)
Chronic pulmonary disease	1.142 (1.04–1.253) *
Rheumatic disease	1.158 (0.934–1.437)
Renal disease	1.283 (1.133–1.454) *
Hepatitis and liver cirrhosis	0.846 (0.774–0.924) *
Alcoholic liver disease	1.041 (0.794–1.365)
Gout	1.034 (0.935–1.143)
Atopic dermatitis	0.963 (0.766–1.209)
ENT allergies	0.978 (0.877–1.091)
Eye disease	
Keratopathy	0.921 (0.784–1.083)
Dry eye syndrome	0.998 (0.859–1.161)
Uveitis	0.995 (0.751–1.318)
Glaucoma	0.951 (0.783–1.156)
Age-related macular degeneration	0.809 (0.604–1.086)
Cataracts	1.181 (1.064–1.312) *
Retinopathy after vitrectomy	0.823 (0.583–1.16)
Concomitant medication	
Systemic steroids	1.194 (1.085–1.314) *
Topical steroids	1.044 (0.941–1.158)
Antipsychotics	1.298 (1.104–1.527) *
Antiarrhythmics	2.072 (1.239–3.465) *
Hypolipidemics	1.081 (0.979–1.193)
Hypoglycemics	0.882 (0.812–0.959) *

* *p*-value < 0.05; aHR: adjusted hazard ratios; NPDR: non-proliferative diabetic retinopathy; DM: diabetes mellitus; DR: diabetic retinopathy; PDR: proliferative diabetic retinopathy.

**Table 6:** Stratified analysis of CVD among all study groups.

	Bonferroni Adjusted aHR (95% C.I.)		
Stratification	DM with DR	Non-DM	DM without DR
Any cerebrovascular or cardiovascular diseases	1.215 (1.068–1.382)	0.573 (0.514–0.639)	Reference
Ischemic heart disease	1.194 (1.004–1.421)	0.614 (0.531–0.710)	Reference
Congestive heart failure	1.915 (1.376–2.665)	0.642 (0.464–0.889)	Reference
Ischemic stroke	2.003 (1.331–3.015)	0.675 (0.462–0.986)	Reference

aHR: adjusted hazard ratios; DM: diabetes mellitus; DR: diabetic retinopathy; Non-DM: non-diabetes mellitus.

**Table 7:** Stratified analysis of the disease, NPDR and PDR.

	Bonferroni Adjusted aHR (95% C.I.)		
Stratification	DM Group	NPDR	PDR
Any CVD	Reference	0.940 (0.790–1.118)	1.612 (1.361–1.909)
Ischemic heart disease	Reference	0.965 (0.765–1.218)	1.545 (1.231–1.939)
Congestive heart failure	Reference	0.982 (0.592–1.628)	3.482 (2.334–5.195)
Ischemic stroke	Reference	1.395 (0.785–2.479)	3.802 (2.261–6.394)

aHR: adjusted hazard ratios; DM: diabetes mellitus; NPDR: non-proliferative diabetic retinopathy; PDR: proliferative diabetic retinopathy; CVD: cardiovascular disease.

## Data Availability

The data presented in this study are available on request from the corresponding author.
